# The role of novel instruments of brand communication and brand image in building consumers’ brand preference and intention to visit wineries

**DOI:** 10.1007/s12144-021-02656-w

**Published:** 2022-01-07

**Authors:** Mar Gómez-Rico, Arturo Molina-Collado, María Leticia Santos-Vijande, María Victoria Molina-Collado, Brian Imhoff

**Affiliations:** 1 Department of Business Administration, University of Castilla-La Mancha, Toledo, Spain; 2 Department of Business Administration, CUNEF University, Madrid, Spain; 3 Department of Hispanic Studies, Texas A&M University, College Station, TX USA

**Keywords:** Brand communication, Brand image, Brand preference, Consumers, Intention to visit, Wine tourism

## Abstract

This research aims to analyze brand communication and brand image as specific drivers of wine brand preference and their influence on wine consumers’ intention to visit associated wineries. Specifically, this paper enhances the understanding of the roles of advertising-promotion, sponsorship-public relations, corporate social responsibility, and social media in brand communication, as well as functional, emotional and reputation components in brand image development in the context of wine tourism industry. Data was collected through a structured and self-administered questionnaire from 486 visitors to wineries in Spain. Partial least squares regression was used to evaluate the measurement model and the hypotheses. The empirical analysis shows that brand communication and brand image have similar positive effects on brand preference, and that brand image mediates the relationship between brand communication and brand preference. This research suggests implications for theory and practice relative to brand management in terms of communication and image; and it proposes insights into novel communication tools and marketing activities for the winery tourism industry. Firms should employ a holistic evaluation of brand communication to involve the whole organization, which would enhance the strategic role that brand communication plays.

**Supplementary Information:**

The online version contains supplementary material available at 10.1007/s12144-021-02656-w.

## Introduction

Wine tourism is a young industry that is rapidly growing and one that represents a significant commercial and financial interest for wineries. Although wineries utilize many mechanisms to attract potential consumers and to increase in-person visits, some of these mechanisms are changing. Therefore, it is crucial to understand the factors underlying visits to wineries (Byrd et al., 2016; Gu et al., 2020), especially since they have decreased significantly due to the COVID-19 pandemic (Wittwer, 2020). The factors underlying visits to wineries addresses a primary gap in the literature that the current research seeks to fill by applying a branding model involving a core wine product. When the consumer prefers a particular brand of wine, the probability of that consumer visiting the winery where it is produced increases (Gómez et al., 2015). Thus, one should consider how the interface between consumers and wineries can be improved to meet or increase consumers’ brand preferences. Sales of core products like wine can be increased by improving key elements, such as branding. Strengthening a brand may be the best way to achieve the above-mentioned goal (Aaker, 1997; Zehir et al., 2011).

A brand is one of the most valuable assets of any firm that wishes to develop competitive advantages (Schultz et al., 2013). Brands are relevant in consumer markets where firms seek to develop trust, loyalty, and consumer preferences. Consumer brand preference is a critical measure for understanding consumer choice behavior and its influence on purchase or visit intention (Ebrahim et al., 2016; Ye et al., 2017). Building strong brands improves brand preference, so how a brand is communicated, and its image strengthened, may influence brand preference (Ebrahim et al., 2016; Schultz et al., 2013). Brand communication is central to transforming product value into consumer behavior (Dolan & Goodman, 2017). Given the competitive challenges faced by most companies, developing marketing strategies that utilize brand communication is crucial to achieving organizational objectives (Luxton et al., 2015). Most prior studies focused on the communication process, which is defined as integrated marketing communications (IMC), instead of on individual components (Finne & Grönroos, 2017; Madhavaram et al., 2005). In fact, relatively little empirical research has been undertaken to examine the main elements that comprise brand communication (Dwivedi & McDonald, 2018). Marketing Science Institute (MSI) 2020–22 research priorities clarify that it is critical to use effective tools for communication in order to understand how factors vary for younger generations (Marketing Science Institute 2020). This paper improves the understanding of the components of brand communication in the context of wine tourism in which digital and social activities have been increased, especially due to the COVID-19 pandemic.

Through a review of frameworks, several dimensions of brand communication have been identified that rely on traditional instruments, such as advertising or sponsorship (Buil et al., 2013; Zehir et al., 2011) and social media (Finne & Grönroos, 2017; Godey et al., 2016), as well as novelty components such as corporate social responsibility (CSR) (Türkel et al., 2016). Despite the importance of these elements for branding, scholars highlight the need to further examine the effects of these instruments (Godey et al., 2016). Moreover, since each communication mode has its own characteristics, it is necessary to maintain a balanced approach. Notably, few studies have considered the role of CSR in brand communication (Türkel et al., 2016).

Brand image increases product memory and has several advantages for business expansion (Keller, 1993; Martínez & Pina, 2009). For vineyards, brand preference and, consequently, visit intention are linked to a wine’s quality, attractiveness, and reputation. The variety of wine is also an essential attraction for wine tourists (Bruwer & Buller, 2013). Thus, a well-communicated brand image is important for improving a brand’s position in market competition. Brand communication strategies are crucial for developing a reputation and the image perceived by consumers.

While previous studies have examined the determinants of winery visits (Gómez et al., 2019), most have focused on New-World wine countries (e.g., USA, New Zealand, Australia). Therefore, an opportunity exists to undertake strategic research identifying the drivers of winery visits within Old-World wine-producing countries (e.g., Spain, Italy, France) in the context of branding (Gómez et al., 2019).

The aim of this paper is to analyze the novel instruments of brand communication and brand image as specific drivers of wine brand preference and their influence on wine consumers’ intention to visit associated wineries. The specific purposes are to cover gaps in background literature and, more specifically: (1) to develop and empirically test a model based on wine as a primary attraction in the wine tourism context; (2) to examine and assess which brand communication strategies works best with wine tourists, who are important to the praxis of branding (Braun et al., 2014); (3) to measure brand communication as a multidimensional concept with conventional, digital and social measures; (4) to measure brand image as a multidimensional construct based on tangible and intangible dimensions; and (5) to incorporate into scholarly literature on winery tourism the Old-World wine-producing country of Spain, which ranks as the top destination in Europe for wine tourism.

The structure of this research is organized as follows. First, we present the definitions of the constructs of interest that are considered for the development of the model. Then, we describe the method and results of the survey and hypotheses tests. The last section presents the conclusions, implications, limitations, and suggestions for future studies.

## Literature Review

### Wine Brand Communication

Brand communication is based on the idea that any product or service can be marketed by using elements that distinguish it from those of competitors. Brand communication promotes connections between companies and customers because consumers buy brands that they remember the most (Madhavaram et al., 2005). It is the most relevant element for effectively introducing a new product or service into a market (Schultz et al., 2013).

Brand communication is also a strategic tool that helps companies be more effective in achieving their communication goals. Good brand communication must be driven by responsibility and incorporate both traditional mediums and emerging networks in order to enhance a firm’s competitive advantage (Luxton et al., 2015). Given the incredible growth of social media and its rapid evolution, the advertising environment has led to relevant changes in brand communication theory and practice that highlight the importance of integrating social media and its strategic role (Madhavaram et al., 2005).

Both unidimensional and multidimensional measures have been used for brand communication. Some researchers have conceptualized it as a unidimensional result of both advertising and promotions (Grace & O’Cass, 2005; Zehir et al., 2011). However, the more widespread form of measurement is multidimensional, typically as the sum of two main dimensions: one-way or indirect communication (i.e., print, TV, radio); and two-way or direct communication (Azize 2012). Much of the relevant literature suggests that the main dimensions of brand communication are advertising, sponsorship, and various alternative communication options (Keller, 2003; Madhavaram et al., 2005). Porcu et al. (2017) suggest that brand communication can be measured by the following dimensions: public relations, advertising, direct sales, and promotion.

It is generally accepted that marketers attempt to coordinate offline and online communications comprised mainly of social media (Batra & Keller, 2016). Some authors suggest that messages should be communicated through consistent advertisements, sponsorships, and digital media, as each communication option addresses a different objective. Other researchers highlight the importance of considering the social perspective as multidimensional in the study of brand communication. CSR has emerged as a commonly used marketing tool, since customers view a company more favorably if it promotes social causes (Nan & Heo, 2007; Simmons & Becker-Olsen, 2006). In the actual interactive society, CSR is considered a form of communication (Schultz et al., 2013).

In this research, brand communication is conceptualized as a four-dimensional construct that balances the following traditional and online media elements, including social aspects of them: advertising promotions, sponsorship-public relations, social media and CSR (Dwivedi & McDonald, 2018). These dimensions have been studied individually through customer assessments of brand communication attributes in previous research, i.e.: for advertising promotions (Zehir et al., 2011), for sponsorship-public relations (Chung et al., 2013; Fortunato, 2009), for social media activities (Voorveld, 2019) and for CSR (Nan & Heo, 2007; Westberg & Pope, 2014). Next, we briefly explain the basic features of these four dimensions.

First, advertising and promotions are of particular interest in marketing and consumer behavior (Buil et al., 2013; Grace & O’Cass, 2005; Kwon et al., 2020; Lee et al., 2020). Advertising is a powerful tool for communicating a brand’s functional and emotional value, so it must be appropriately designed and executed to contribute to the creation of a strong brand (Aaker, 1997). Promotion is a mode of advertising intended to increase the sales of a product or service. Advertising can therefore be conceptualized in terms of promotion campaigns and memorability. Many researchers categorize both concepts in the same dimension because they are related (Grace & O’Cass, 2005).

Second, sponsorship and public relations are interrelated and employed to achieve management goals. Although sponsorship is considered a type of public relations (Chung et al., 2013), both can communicate a valuable brand association. Firms choose their sponsorships, which can have a positive effect on brand communication, to achieve public relations objectives (Fortunato, 2009), and a natural fit occurs between them when there is congruence in brand messaging. This congruence is derived from the mission, product(s), market segments and attributes of the product(s), among other factors (Simmons & Becker-Olsen, 2006). Sponsorship is a marketing partnership of two or more brands that are visibly joined to a product or a service (Cornwell & Kwon, 2020). Public relations are defined as activities that project a good image of a company to different stakeholders (Chung et al., 2013; Fortunato, 2009).

Third, social media as a category comprises online social media actions that have been determined to be proper and positive (Kim & Ko, 2012; Kwon et al., 2020). Its development has transformed communication theory and includes consumers as active participants in the process of communication that can even create brand content through blogs or online communities (Batra & Keller, 2016; Finne & Grönroos, 2017). Through social media activities that encourage entertainment, interaction, trendiness, customization, and word-of-mouth recommendations, more personal relationships can be created with consumers (Godey et al., 2016; Tarkang et al., 2020). Social media channels represent an alternative way for firms to communicate, and they are less expensive and more effective for certain targets.

Fourth, CSR is used to assess a brand’s responsibility to its community (Nan & Heo, 2007; Westberg & Pope, 2014). Although there is a lack of consensus on the definition of CSR, most scholars agree that it refers to the social role that a company plays in society, and that it can maximize a company’s image through ethical and social interactions. CSR initiatives should be designed to help all stakeholders achieve personal goals and establish an emotional connection and positive relationships with them. CSR has emerged as a commonly used marketing tool, since customers will view a company more favorably if it promotes social causes (Simmons & Becker-Olsen, 2006).

In the context of wine tourism, wine represents the core attraction, and communication activities that promote this activity are truly important. It is essential to analyze how wine brand communication is carried out using different types of communication strategies. The development of brand communication as a multidimensional concept that can be used in wine market is a novel and great challenge for vineyards (Dolan & Goodman, 2017).

### Wine Brand Image

Brand image is defined as a function of how consumers perceive and remember a brand. Brand image is developed by a network of concepts interrelated by associations (Martínez & Pina, 2009), which invokes a set of beliefs about a specific brand that plays a relevant role in a customer’s decision-making process. A customer creates an image from the synthesis of all the symbols used by the brand. Brand image is the result of customers’ interpretations of brand value (Chang & Liu, 2009).

Both qualitative and quantitative methods have been used to measure brand image. The former is frequently applied to explore brand perceptions. Quantitative research employs several types of scales for confirmatory studies (Keller, 1993). Research confirms that brand image has a structure that is more multidimensional than unidimensional (Martínez & Pina, 2009). It is a multifaceted construct (Gómez et al., 2015).

Brand image also includes functional aspects, such as the relationship between consumption and symbolic and experiential needs. According to Biel (1992), brand image consists of three dimensions: corporate image, product/service image and user image. Jara and Cliquet (2012) relate it to tangible attributes (also called functional benefits), symbolic benefits and personality values, among other elements. Following Martínez and Pina (2009), brand image is related to functional image, affective image, and reputation of the brand. These three dimensions have been used to evaluate different products. Thus, in this research, brand image is conceptualized as a multidimensional concept that includes these three dimensions: functional image, affective image, and reputation. Functional image refers to quality requirements that create value. The affective dimension refers to intangible elements that are similar to a personality or a positive association in the customer’s mind. Reputation is the global attitude toward the brand that includes a total evaluation of the company. Reputation refers to an updated image or to distinctive or pervasive elements (Cobb-Walgren et al., 1995).

For wine, brand image is the essence of what wine consumers want or value. Wine brand image is usually associated with quality, and wineries use labels or medals to highlight special. Intangible attributes support the hedonic experience of wine consumption (Bruwer & Rueger-Muck, 2019). Wine brand image is a multidimensional concept involving nature, and it should be studied in depth.

### Wine Brand Preference

Brand preference is usually measured by asking customers to specify their favorite brands from a group of selected products/services (Godey et al., 2016; Keller, 2003). Consumer preference for a brand is a function of consumers’ beliefs about the brand’s attributes (Ebrahim et al., 2016). Brand preference indicates the desire to buy a product or service, even when other products or services have the same price and attributes (Cobb-Walgren et al., 1995). This concept is relevant for organizations because it offers a loyalty indicator and points to marketing success. Brand preference is the bias that a consumer has toward a certain brand (Chang & Liu, 2009). Understanding how wine consumers feel about their favorite brands is an interesting topic to study. Wine brand preference can be affected by wine knowledge, which is obtained through information provided by wineries and the recommendations of other wine consumers (Bruwer & Buller, 2013).

### Intention to Visit, Revisit and/or Recommend Wineries

Intention to visit refers to the likelihood that a traveler will visit a place. This construct is related to consumers’ attitudes and, thus, is a good measurement of how customers evaluate a brand. However, intention to visit may also refer to a consumer’s plan to visit a place or destination (Chang & Liu, 2009). Intentions are different from attitudes. While attitudes consist of a set of evaluative judgements or predispositions, intentions reveal an individual’s motivation or mindful plan to engage in a behavior regarding a brand. In the wine tourism context, intention to visit implies the intention to visit a new winery, the intention to revisit a winery and/or the likelihood of recommending the visited winery to other people (Ye et al., 2017). It is an important behavioral measure that determines the degree of interest in visiting a winery or a wine region.

## Research Hypotheses

### Wine Brand Communication and Wine Brand Preference

Brand communication has the ability to influence brand preference because a brand’s marketing efforts are continually evaluated by consumers. By creating favorable preferences, brand communication generates competitive advantages for companies (Madhavaram et al., 2005). It is a key component in the management of brand relationships with users and with the community at large. Brand communication helps consumers feel more attached to a brand, can strengthen relationships over time, and generates a preference for products (Azize 2012; Grace & O’Cass, 2005; Schultz et al., 2013).

Advertising reflects the willingness to tell the world about a product or service and can be used to project brand personality and ethos (Dwivedi & McDonald, 2018). Advertising and promotion are primary contributors to consumer perceptions, which create value and generate positive brand evaluations that affect attitudes or behaviors toward a brand (Cobb-Walgren et al., 1995; Cornwell & Kwon, 2020). Customer appreciation of brand sponsorship can also make a brand more attractive. A good fit between a firm and its sponsorship activities can lead to the achievement of public relations goals and positively impact brand preference (Cornwell & Kwon, 2020; Fortunato, 2009; Simmons & Becker-Olsen, 2006). It is also the case that CSR impacts brand preference and, consequently, purchase intentions and intention to visit (Polonsky & Jevons, 2009; Türkel et al., 2016). Brands communicate their integrity to society through CSR, which positively promotes brand preference due to firms’ moral authenticity. Social media activities improve brand preference by expanding the interaction between firms and consumers (Canovi & Pucciarelli, 2019).

The brand communication implemented by vineyards directly influences wine preference (Dolan & Goodman, 2017). Visitors search for information to aid in their decision-making about wine tourism destinations. Wineries can differentiate themselves from competitors by gaining a better understanding of how to communicate the attributes of their wine, or by the special activities that they provide (Gu et al., 2020). Any exposure to brand communication affects visitors’ responses, but wine tourists respond differently to brand communication messaging.

Consequently, we propose the following hypothesis 1:

- **H1** Wine brand communication is positively related to wine brand preference.

### Wine Brand Image and Wine Brand Preference

Brand image provides signals that can improve consumer preferences for products or services (Erdem & Swait, 1998), creating considerable advantages that increase brand preference. This result is particularly true of the functional aspects that affect brand preference (Erdem & Swait, 1998). A positive brand image emphasizes unique brand associations (Jara & Cliquet, 2012; Keller, 1993). Brand image also plays a relevant role in the decision-making process of customers (Braun et al., 2014). It is developed through a network of biased evaluations that impact the responses of consumers’ brand preference and, consequently, their intention to buy or to visit (Jara & Cliquet, 2012). Most academics agree that a stronger brand image is correlated with the ability to charge a premium price, with loyalty, and with brand preference (Aaker, 1997; Godey et al., 2016; Keller, 1993).

The quality and variety of wines affect consumer purchase preferences, and they are crucial factors that can positively affect preference for specific brands (Byrd et al., 2016). Wine is more affected by supplementary elements, and the purchase of wine is positively correlated with brand preference (Schultz et al., 2013).

We therefore propose a second hypothesis:

- **H2** Wine brand image influences wine brand preference.

### Wine Brand Communication and Brand Image

Communicating a brand image is an important marketing activity that helps establish a brand’s preference (Luxton et al., 2015; Simmons & Becker-Olsen, 2006). Effective communication helps firms increase brand value through strategic business processes (Madhavaram et al., 2005). Given that brand image is derived from a perception that is influenced by brand communication, a common marketing goal is to develop brand communication strategies that convey a well-coordinated brand image which is crucial for a company’s success. How brand values are communicated directly affects brand image (Chang & Liu, 2009). Communication via traditional activities, or through positive word-of-mouth and physical activities, influences brand image and improves the brand’s success (Braun et al., 2014). Marketing communication strategies allow the market to learn about a brand, creating value that enhances brand image, which can lead to a positional advantage in the market (Luxton et al., 2015).

Traditional and social media communication have an important influence on brand image (Keller, 2003; Madhavaram et al., 2005). Advertising can influence brand image in different ways, since individuals’ attitudes toward advertisements play a relevant role in developing it (Keller, 2003). Advertising and price promotions affect brand image by generating value for brands (Buil et al., 2013). Sponsorship can also enhance brand image effectively, though sponsorships that are not a “good fit” can dilute brand image (Madhavaram et al., 2005; Simmons & Becker-Olsen, 2006). Although public relations, like sponsorship, can both enhance brand image and generate different outputs whose respective effectiveness can be measured by brand image (Chung et al., 2013; Cornwell & Kwon, 2020; Fortunato, 2009). CSR attracts consumers, strengthens relationships with stakeholders, motivates employees and improves the image and reputation of brands (Polonsky & Jevons, 2009). Social media marketing efforts have an influence on the two main dimensions of brand equity: awareness and image (Canovi & Pucciarelli, 2019; Kim & Ko, 2012).

Perceptions of wine brand image are influenced by communicated messages, which play a relevant role in the wine market (Dolan & Goodman, 2017). Wineries communicate to consumers using different methods that directly affect wine brands. Visitors will prefer the brand that has the highest recall (Bruwer & Rueger-Muck, 2019).

Consequently, we propose a third hypothesis:

- **H3** Wine brand communication positively affects wine brand image.

### Wine Brand Preference and Wine Tourism Intention to Visit, to Revisit and/or to Recommend

Purchase intention is influenced by brand preference, the effective development of which leads to an increase in purchasing (Chang & Liu, 2009; Cobb-Walgren et al., 1995). Brand preference is a fundamental aspect of consumer choice behavior (Gu et al., 2020). It is a crucial element of a successful destination brand and can enhance the intention to visit that destination. Intention to visit correlates strongly with brand preference. The greater the preference is for a wine brand, the greater will be the interest in visiting the winery (Baalbaki & Guzmán, 2016). In the wine market, the wine, brand, and preference are intercorrelated with intention to visit (Byrd et al., 2016).

Our fourth and final hypothesis follows:

- **H4** Wine brand preference influences the intention to visit wineries.

**Fig. 1:**
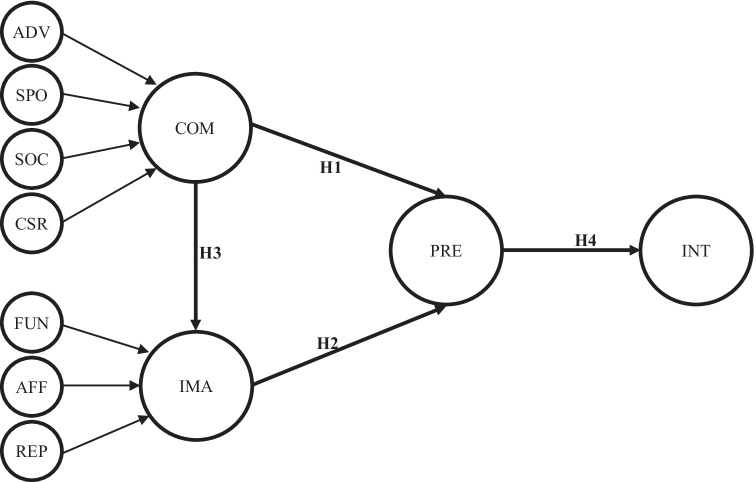
Theoretical model. Notes: ADV: advertising-promotion; SPO: sponsorship-public relations; SOC: social media; CSR: corporate social responsibility; FUN: functional image; AFF: affective image; REP: reputation; COM: wine brand communication; IMA: wine brand image; PRE: wine brand preference; INT: intention to visit, revisit and/or recommend wineries

The wine brand tourism model that follows illustrates the elements that precede and determine intention to visit wineries and the factors that feed those elements (Fig. 1):

## Method

### Data Collection and Sample

The research method used to achieve the objectives of this work is detailed below. Using a structured self-administered survey questionnaire, quantitative data were collected in situ from visitors to wineries in Spain. Of 486 total respondents, 47.4% were male, 52.6% were female, and most fell between the ages of 25 and 54 (77.8%). The majority of respondents were employed (62.6%), had completed university studies (57.3%), and were married or living as a couple (69.9%). In terms of household income, 37.4% declared they had a total monthly income higher than 3,000€.

### Measures

The survey instrument included two major categories: (1) sociodemographic data; and (2) rating scales regarding brand communication, brand image, brand preference and intention to visit. The instrument rating scales were created based on information from current background literature (Table 1). Brand communication was measured using a 16-item scale proposed by Dwivedi and McDonald’s (2018) that was adapted to the wine sector. This instrument considers the following dimensions: advertising promotion, sponsorship-public relations, social media, and CSR, which are consistent with the results of our content analysis. Brand image was assessed using Martínez and Pina’s (2009) 8-item scale that considers three dimensions: functional image, affective image, and reputation. Finally, Ebrahim et al.’s (2016) 3-item scale was used to measure brand preference, and Byrd et al.’s scale (2016) was used to assess intention to visit. We used a 7-point Likert scale for all the items. Brand communication and brand image were conceptualized as higher-order reflective-formative constructs. Accordingly, brand communication is formatively measured across four reflective dimensions (advertising promotion, sponsorship-public relations, social media, and CSR); and brand image is formatively measured across three reflective dimensions (functional image, affective image, and reputation). A reflective latent construct entails that changes in the underlying latent construct are reflected by changes in the indicators, i.e., reflective measures are caused by the latent construct (Jarvis et al., 2003). Formative constructs mean that the measures cause the construct and that the construct is fully derived by its measurement. Therefore, an improvement in the underlying latent construct would not require a simultaneous increase in all of the indicators (Bollen & Lennox, 1991). An improvement of brand communication (COM) can be achieved with a single improvement of any of its dimensions (advertising, sponsorship-public relations, or social media). Similarly, a better brand image (IMA) can be attained with different combinations of reputation and functional and affective image. In other words, an improvement in COM or IMA does not necessarily imply an improvement in all its constituent dimensions and, therefore, they are not considered reflective constructs. Similarly, as opposed to reflective indicators, formative indicators are not interchangeable because the construct is a weighted, linear combination of all its observed measures. The constituent dimensions of brand communication and brand image represent different concepts, and eliminating a formative indicator from the measurement model would change the meaning of the composite construct (Coltman et al., 2008).

**Table 1:** Measurement scales

Constructs/Dimensions	Items	Sources	
Wine brand communication (COM)	ADV	ADV1. I react favorably to the advertising and promotions of this wine brand	Buil et al. (2013); Chung et al. (2013); Dwivedi and McDonald (2018); Fortunato (2009); Godey et al. (2016); Grace and O’Cass (2005); Schivinski and Dabrowski (2015); Türkel et al. (2016); Westberg and Pope (2014); Zehir et al. (2011)
		ADV2. I feel positive towards the advertising and promotions of this wine brand	
		ADV3. The advertising and promotions of this wine brand are good	
		ADV4. I like the advertising and promotions of this wine brand	
	SPO	SPO1. The wine brand’s sponsorship and public relations activities are good	
		SPO2. I find this wine brand’s sponsorship and public relations activities are very positive	
		SPO3. The wine brand’s sponsorship and public relations activities are very appealing	
		SPO4. I am favorably disposed towards this wine brand’s sponsorship and public relations activities	
	SOC	SOC1. Social media communications for this wine brand meets my expectations	
		SOC2. Social media communications for this wine brand are very attractive	
		SOC3. I am satisfied with social media communications for this wine brand	
		SOC4. Social media communications for this wine brand perform well, when compared with social media communications of other companies	
	CSR	CSR1. This wine brand believes in philanthropy and giving generously to worthy causes	
		CSR2. This wine brand is genuinely concerned about consumer welfare	
		CSR3. This wine brand is highly concerned about environmental issues	
		CSR4. This wine brand is highly involved in community activities	
Wine brand image (IMA)	FUN	FUN1. The wines have a high quality	Gómez et al. (2015); Martínez and Pina (2009)
		FUN2. The wines have better characteristics than competitors	
		FUN3. The wines of the competitors are usually cheaper	
	AFF	AFF1. This wine brand is nice	
		AFF2. This wine brand has a personality that distinguishes itself from competitors	
		AFF3. It’s a wine brand that doesn’t disappoint its customers	
	REP	REP1. It’s one of the best wine brands in the sector	
		REP2. This wine brand is very consolidated in the market	
Wine brand preference (PRE)	PRE	PRE1. I like this brand more than any other brand of wines	Ebrahim et al. (2016); Chang and Liu (2009); Cobb-Walgren et al. (1995); Godey et al. (2016)
		PRE2. This is my preferred brand over any other brand of wines	
		PRE3. When it comes to making a purchase, this brand of wines is my first preference	
Intention to visit, revisit and/or recommend wineries (INT)	INT	INT1. I will visit this winery again	Byrd et al. (2016); Chang and Liu (2009); Cobb-Walgren et al. (1995)
		INT2. The likelihood of my revisiting this winery is high	
		INT3. I will recommend visiting this winery	

Notes: ADV: advertising-promotion; SPO: sponsorship-public relations; SOC: social media; CSR: corporate social responsibility; FUN: functional image; AFF: affective image; REP: reputation

### Data Analysis

We used partial least squares structural equations modeling (PLS-SEM) to examine the accuracy of the scales and the model (Chin, 2010; Henseler et al., 2016). The literature supports that PLS-SEM allows the use of formative and reflective indicators, therefore using different measurement modes, and is especially appropriate for second-order measurement models (Ringle et al., 2012). Thus, the proposed model includes two reflective unidimensional constructs: brand preference and intention to visit (we used Mode A measurement model); and two higher-order constructs: brand communication and brand image (Mode B measurement). The latter involves examining two second-order reflective-formative models. In this study, to specify the higher-order measurement models, we used the two-step approach described by Becker et al. (2012). This method estimates the construct scores of the first-order constructs in a first-stage model without the second-order construct presented and, subsequently, uses the first-stage latent variable construct scores obtained as indicators for the higher-order latent variable in a separate second-stage analysis (Becker et al., 2012). In this case, Mode B of measurement is used for the formative construct and Mode A for the reflective constructs (Becker et al., 2012; Wetzels et al., 2009).

### Treatment of Common Method Bias

Using a single informant to obtain information from exogenous and endogenous constructs can be a source of common method bias as participants may develop a specific pattern answering. Many other factors, such as social desirability, ambiguous wording, and scale length, can affect how the respondent replies to questions, thereby resulting in method biases. Podsakoff et al. (2003) identify two primary ways to control for method biases: procedural and/or statistical remedies. Procedural remedies attempt to minimize common method bias through study design. In this sense, the questionnaire design included a psychological separation between the predictor and criterion variables to avoid any direct connection between their measurement. Procedural remedies also included ensuring the anonymity of respondents, emphasizing that there are no right or wrong answers, and improving the wording of items by pre-testing the questionnaire with four academics prior to its launch. Furthermore, we used Harman’s one-factor test to statistically test whether common method bias can be a problem in this study (Podsakoff et al., 2003). In this test, evidence for common method bias exists when a single factor emerges from the analysis or when one general factor accounts for most of the covariance in the independent and dependent variables.

The factorial analysis carried out shows that the variance explained for the first factor is 42.016%, with principal components as the method of extraction (Varimax rotation), which is below the 50% value threshold. Furthermore, the number of factors in the unrotated factor structure is seven. These seven factors are equivalent to a total variance explained of 75.215%. However, Harman’s single-factor test has limitations, principally referred to an insufficient sensitivity to detect moderate or small levels of common method bias effects (Malhotra et al., 2006).

To address this problem, we used the marker variable technique described by Lindell and Whitney (2001). This technique requires researchers to identify a marker variable theoretically unrelated to at least one of the other variables in the model (Simmering et al., 2015). Because the marker variable is assumed to have no relationship with one or more variables in the study, common method bias can be assessed based on the correlation between the marker variable and the theoretically unrelated variables (Malhotra et al., 2006). To apply Lindell and Whitney’s (2001) technique, the extent to which respondents “*like to know what products or services make a good impression in other individuals*” was chosen as a theoretically unrelated marker variable. The average correlation between the marker variable and the constructs of the structural equation model was 0.008, and the average significance was 0.549 (two-tailed), which is above the thresholds of 0.05 (two-tailed) necessary to consider correlations as being significant. When this marker variable was included in the structural equation model, none of the dependent variable variances significantly increased. Moreover, the impact of the marker variable on the model’s endogenous variables is not significant. In conclusion, we understand that common method bias was not a problem in this study.

## Results

### Evaluation of the Measurement Model

The first stage of the analysis allows evaluating the psychometric properties of the scales. Analysis of the saturated model through SRMR (standardized root mean square residual) was used to determine model fit (Henseler et al., 2016). Following the work of Hu and Bentler (1999) it presents an appropriate cutoff value (below of 0.08). After that, the evaluation of the composite reliability, Cronbach’s alpha (α), and average variance extracted (AVE) was developed (Table 2). Finally, Fornell and Larcker’s (1981) test and the HTMT criterion were used to verify discriminant validity (Table 3). All the measures present adequate convergent validity and reliability.

**Table 2:** Measurement model evaluation

Constructs/Dimensions		Items	Factor loadings (t bootstrap)	Cronbach’s α	rho	CR	AVE
Wine brand communication (COM)	ADV	ADV1	0.787*** (36.116)	0.823	0.830	0.883	0.653
		ADV2	0.838*** (54.900)				
		ADV3	0.823*** (45.731)				
		ADV4	0.782*** (34.276)				
	SPO	SPO1	0.856*** (61.991)	0.874	0.881	0.913	0.724
		SPO2	0.850*** (58.845)				
		SPO3	0.859*** (54.656)				
		SPO4	0.837*** (48.016)				
	SOC	SOC1	0.909*** (97.412)	0.926	0.928	0.947	0.818
		SOC2	0.862*** (60.375)				
		SOC3	0.920*** (101.026)				
		SOC4	0.927*** (103.059)				
	CSR	CSR1	0.960*** (189.496)	0.953	0.955	0.966	0.878
		CSR2	0.955*** (171.595)				
		CSR3	0.918*** (102.333)				
		CSR4	0.913*** (108.034)				
Wine brand image (IMA)	FUN	FUN1	0.915*** (111.323)	0.851	0.864	0.910	0.771
		FUN2	0.871*** (53.911)				
		FUN3	0.846*** (52.275)				
	AFF	AFF1	0.917*** (99.888)	0.850	0.864	0.909	0.768
		AFF2	0.837*** (39.348)				
		AFF3	0.874*** (57.870)				
	REP	REP1	0.923*** (126.680)	0.768	0.796	0.895	0.809
		REP2	0.875*** (54.229)				
Wine brand preference (PRE)	PRE	PRE1	0.938*** (145.369)	0.850	0.859	0.910	0.773
		PRE2	0.907*** (84.727)				
		PRE3	0.785*** (31.649)				
Intention to visit, revisit and/or recommend wineries (INT)	INT	INT1	0.945*** (142.950)	0.921	0.926	0.951	0.865
		INT2	0.968*** (224.060)				
		INT3	0.875*** (47.125)				

Notes: ***p < 0.001; ADV: advertising-promotion; SPO: sponsorship-public relations; SOC: social media; CSR: corporate social responsibility; FUN: functional image; AFF: affective image; REP: reputation; CR: composite reliability; AVE: average variance extracted

**Table 3:** Measurement model. Discriminant validity

Fornell-Larcker									
	ADV	AFF	CSR	FUN	INT	PRE	REP	SOC	SPO
ADV	**0.808**								
AFF	0.509	**0.877**							
CSR	0.468	0.584	**0.937**						
FUN	0.578	0.472	0.473	**0.878**					
INT	0.516	0.379	0.380	0.455	**0.930**				
PRE	0.477	0.381	0.250	0.419	0.470	**0.879**			
REP	0.645	0.486	0.489	0.553	0.393	0.387	**0.900**		
SOC	0.664	0.355	0.379	0.451	0.375	0.406	0.537	**0.905**	
SPO	0.640	0.462	0.445	0.507	0.409	0.380	0.706	0.522	**0.851**
**Heterotrait-Monotrait ratio (HTMT)**									
	ADV	AFF	CSR	FUN	INT	PRE	REP	SOC	SPO
ADV									
AFF	0.594								
CSR	0.523	0.645							
FUN	0.682	0.550	0.521						
INT	0.584	0.425	0.405	0.516					
PRE	0.563	0.441	0.278	0.491	0.532				
REP	0.801	0.592	0.564	0.674	0.460	0.475			
SOC	0.761	0.398	0.401	0.502	0.405	0.455	0.632		
SPO	0.749	0.525	0.485	0.576	0.453	0.434	0.843	0.577	

Notes: ADV: advertising-promotion; SPO: sponsorship-public relations; SOC: social media; CSR: corporate social responsibility; FUN: functional image; AFF: affective image; REP: reputation; PRE: wine brand preference; INT: intention to visit, revisit and/or recommend wineries. Fornell-Larcker: The values on the diagonal are the square root of the AVEs; and the values on off-diagonal are the correlation between the constructs

### Structural Model

The structural model was estimated for the second-stage by using bootstrapping (10,000 resamples), and t-statistics, and confidence intervals. All four dimensions of brand communication contribute positively to form its multidimensional nature in varying degrees: advertising promotion most strongly (β = 0.454, p < 0.001), followed by sponsorship-public relations (β = 0.366, p < 0.001), CSR (β = 0.270, p < 0.001), and lastly social media (β = 0.131, p < 0.001). Concerning brand image: reputation has the highest influence (β = 0.540, p < 0.001), functional image is the second (β = 0.346, p < 0.001), and affective image is the last one (β = 0.324, p < 0.001).

The VIF of the formative constructs was tested; all results were lower than 3.3. The R^2^ for brand image is 67.6%; 24.9% for brand preference (12.2% of brand the variance in brand communication and 12.7% of the variance in brand image); and 22% for intention to visit. All these values are high or moderate (< 0.75 or < 0.5, respectively) (Hair et al., 2017). Regarding f^2^, there is a weak effect (from 0.02 to 0.15) between brand communication and brand preference (0.028) and between brand image and brand preference (0.031); a large effect (higher than 0.35) between brand communication and brand image (2.089); and a medium effect (from 0.15 to 0.35) between brand communication and brand image (0.285) (Cohen 1992) (Table 4).

**Table 4:** Structural model results

Second-order formative constructs	Estimate	T-value	VIF						
COM—Wine Brand Communication									
Advertising-promotion	0.454	7.376	2.365						
Sponsorship-public relations	0.366	5.981	1.816						
Social media	0.131	2.595	1.851						
Corporate social responsibility	0.270	5.739	1.349						
IMA—Wine Brand Image									
Functional image	0.346	8.124	1.562						
Affective image	0.324	7.052	1.420						
Reputation	0.540	10.909	1.591						
**Hypothesized path**	**Estimate**	**T-value**	**Contrast**	**R**^**2**^** / R**^**2**^** adjusted**	**F**^**2**^	**Mean**	**5%**	**95%**	
**H1**	COM → PRE	0.257**	2.940	Do not reject	24.9% / 24.6%	0.028	0.262	0.117	0.404
**H2**	IMA → PRE	0.267***	3.373	Do not reject		0.031	0.264	0.135	0.395
**H3**	COM → IMA	0.822***	51.797	Do not reject	67.6% / 67.6%	2.089	0.823	0.795	0.848
**H4**	PRE → INT	0.471***	11.064	Do not reject	22.2% / 22.0%	0.285	0.470	0.399	0.538

Notes: COM: wine brand communication; IMA: wine brand image; PRE: wine brand preference; INT: intention to visit, revisit and/or recommend wineries; t (0.05; 4999) = 1.645*; t (0.01; 4999) = 2.327**; t (0.001; 4999) = 3.092***; *p < 0.05; **p < 0.01; ***p < 0.001

The results indicate that brand communication has a positive and significant influence on brand preference; thus, H1 is supported (β = 0.257, p < 0.01, t = 2.940). Brand image has a positive and significant influence on brand preference; thus, H2 is supported (β = 0.267, p < 0.001, t = 3.373). Brand communication exerts positive influence on brand image; thus, H3 is supported (β = 0.822, p < 0.001, t = 51.797). That is, brand communication has an indirect influence on brand preference through brand image, which mediates the relationship between brand communication and brand preference. This indirect effect (brand communication brand image brand preference) is positive and significant (β = 0.219, p < 0.001, t = 3.369). VAF (Variation account for) was used to determine the indirect effect, resulting in a value of 0.46, which confirms partial mediation (0.2 ≤ VAF ≤ 0.8) (Hair et al., 2017). Thus, this model identifies brand image as a mediator. Finally, brand preference exerts a significant positive influence on intention to visit; thus, H4 is supported (β = 0.471, p < 0.001, t = 11.064). Confidence intervals confirm the significance of the results (Henseler et al., 2016) (Table 4).

Table 5 presents the values of Q^2^ (cross-validated redundancy index), with strong predictive relevance for the cases of brand image (0.513) and brand preference (0.490), and moderates for intention to visit (0.218) (Hair et al., 2017).

**Table 5:** PLS predict assessment

Variable Prediction Summary										
	Q^2^									
IMA	0.513									
INT	0.218									
PRE	0.490									
Indicator Prediction Summary										
		**PLS**			**LM**			**PLS-LM**		
		**RMSE**	**MAE**	**Q**^**2**^	**RMSE**	**MAE**	**Q**^**2**^	**RMSE**	**MAE**	**Q**^**2**^
AFF		0.771	0.610	0.408	0.803	0.615	0.358	-0.032	-0.005	0.051
FUN		0.779	0.618	0.396	0.780	0.618	0.395	-0.001	-0.000	0.001
INT1		0.819	0.655	0.200	0.847	0.699	0.144	-0.028	-0.043	0.056
INT2		0.876	0.679	0.246	0.921	0.725	0.167	-0.044	-0.046	0.079
INT3		0.875	0.671	0.277	0.938	0.736	0.168	-0.064	-0.065	0.109
PRE1		1.125	0.906	0.231	1.140	0.915	0.211	-0.015	-0.009	0.020
PRE2		1.024	0.809	0.161	1.029	0.811	0.152	-0.005	-0.001	0.008
PRE3		1.184	0.911	0.140	1.191	0.918	0.130	-0.007	-0.007	0.010
REP		0.654	0.501	0.575	0.665	0.513	0.560	-0.011	-0.012	0.015

Notes: FUN: functional image; AFF: affective image; REP: reputation; PRE: wine brand preference; INT: intention to visit, revisit and/or recommend wineries

## Discussion and Conclusions

Wine tourism is a fast-growing sector and a financially lucrative type of tourism, notwithstanding recent COVID-19 related decreases in winery visits (Wittwer, 2020). As the methods used to attract tourists to wineries evolve, it is important to understand the factors that generate wine brand preference because it directly impacts winery visits (Gu et al., 2020). The aim of this paper was to analyze novel instruments used for wine brand communication and wine brand image as specific determinants of wine brand preference and its influence on wine tourists’ intention to visit wineries. This research contributes to the fields of brand and wine tourism by proposing and testing a model of core wine products and important antecedents, providing evidence of the link between wine and wine tourism and, thereby, addressing a gap in the literature on wine tourism by deepening the understanding of relevant marketing factors that affect the inflow of visitors to wineries.

The results have theoretical and practical implications. The present study contributes to marketing literature in several ways. First, this paper provides insights on the drivers of wine brand preference and intention to visit in the context of wine tourism. To attract repeat visitors to wineries, marketing efforts should be strongly wine-related (Bruwer & Rueger-Muck, 2019). Unlike traditional studies that focus on wine tourism services, this research contributes to theory by shedding light on the relationship between wine brand preference and wine tourism, expanding its nomological network and highlighting novel drivers of brand preference. This study takes a holistic approach to understanding intention to visit wineries and provides empirical evidence on the relationships between brand communication, brand image, brand preference and intention to visit, hence strengthening the theoretical foundation in wine tourism. Moreover, this study was conducted in Spain, which is an Old-World wine-producing country and the top destination in Europe for wine tourism with well over 4000 wineries. Most previous studies that analyze factors of visits to wineries were conducted in New-World wine countries (Gómez et al., 2019).

Second, this study contributes to the field of brand communication as a multidimensional concept that can be adapted to the wine sector. It identifies the four elements of brand communication: advertisement promotion, sponsorship-public relations, social media, and CSR (Buil et al., 2013; Dwivedi & McDonald’s, 2018). These components present a full perspective, as they include controlled and uncontrolled communication media, one-way and two-way communication, and traditional and digital media (Batra & Keller, 2016). Most studies on communication focus on IMC (Porcu et al., 2017), which refers to the integration or coordination of communication activities without considering what the main actions should be in individual companies or sectors. This study focuses on communication actions within traditional and digital media, as well as concepts that have a great impact on companies’ reputations, such as CSR (Türkel et al., 2016). Most previous scholarship focused on narrow instruments that captured only some aspects (Luxton et al., 2015). This research highlights that social media provides numerous opportunities for enhancing brand preference and brand image, and suggests that managers should incorporate the use of social media into their daily communication activities (Schivinski & Dabrowski, 2015). The findings suggest that advertising promotion and sponsorship-public relations play important roles in brand communication and, consequently, in brand preference and brand image (Buil et al., 2013). Therefore, this study provides a holistic and novel evaluation of brand communication. Firms should use an approach that involves the whole organization, which will enhance the strategic role of brand communication (Luxton et al., 2015). The scale used in this study can serve as a tool for evaluating brand communication for managers to analyze the different communication actions used in their wineries.

Third, this work provides new insights into the definition and measurement of brand image by validating Martínez and Pina’s (2009) scale, which considers three dimensions: functional image, affective image, and reputation. The empirical application of this scale shows that brand image can be measured by considering cognitive and emotional information that is linked to psychological responses (Ebrahim et al., 2016). In prior work, brand image had been measured using unidimensional measures (Baalbaki & Guzmán, 2016). The multidimensional scale used in this research represents one of the few endeavors to frame and empirically assess brand image as a combination of tangible and intangible elements, providing quantifiable evidence of their impact on brand preference. The findings indicate that reputation is the main dimension of brand image, followed by functional image and emotional image, in contrast to previous studies that highlight the role of emotional attributes as the main contributors. Functional components, such as wine quality, also have an important role in brand creation (Byrd et al., 2016).

Fourth, the review of literature shows that one of the main limitations for the development of scales is confusion about the definitions and measurement of concepts, which are used in a reflective and formative way, though some authors warn about problems derived from an inadequate specification (Podsakoff et al., 2003). Most studies employ reflective measures without considering that formative measures may be able to improve their specification. In this study, after a rigorous analysis of their conceptualization, brand communication and brand image were measured with formative measures.

Fifth, wine tourists perceive that brand preference results from brand communication and brand image. This research follows previous studies in conceptualizing brand preference (e.g., Ebrahim et al., 2016). The results support the hypotheses and provide evidence of the direct and indirect effects of brand communication. We can confirm that the effects of brand communication on brand preference are aligned with scholarly literature (e.g., Grace & O’Cass, 2005). The results show that tourists who perceive that wineries engage in more communication activities have a greater preference for their wines. In addition, this research provides evidence of the positive influence of brand image on brand preference, which is in line with previous work (e.g., Cobb-Walgren et al., 1995). A positive and strong brand image can increase brand preference and attract and retain wine tourists. The findings suggest that a greater commitment to improving functional and emotional components of wine brand reputation will enhance tourists’ preferences for those wines. Brand image has an important role in transferring inherent value to brand preference. Moreover, the results regarding the indirect effect of brand communication on brand preference by means of brand image are in line with the findings of previous studies (e.g., Godey et al., 2016). The model proposed in this study can be used to determine the variables that have a direct and indirect influence on brand preference. A conceptual model of wine tourism is proposed that includes wine brand preference, and both relationships coexist in this model. That is, brand communication impacts brand preference directly and indirectly by means of brand image. The results suggest that an increase in wine brand preference through brand communication and brand image has important implications for wineries’ brand activities, including communication and actions taken to improve the wineries’ image.

Finally, consistent with Gu et al. (2020), this study provides knowledge about the impact of wine brand on the intention to visit wineries. Intention to visit is conceptualized following previous studies. This research contributes to the literature on wine tourism by assessing the relationship between brand preference and intention to visit. Wineries must pay special attention to how they communicate about their wines and the image they project to tourists. An improvement in the brand preference of their wines impacts not only product sales but also another crucial source of income for wineries, namely, wine tourism.

### Practical Implications

This study provides a valuable tool that wine tourism managers can use to attract tourists to wineries. Consequently, this research makes several recommendations for managers wanting to improve the marketing plan in their wineries. Managers can enhance brand preference by managing two important aspects: brand communication and brand image. They should increase their understanding of both concepts to achieve positive brand outcomes.

First, it is vital to fully understand brand communication from an integrative perspective. In order to develop effective brand campaigns, managers should continue to use traditional advertising and promotions, and reinforce sponsorship and public relations, whose effects persist over a longer period (Simmons & Becker-Olsen, 2006). Attending national and international trade fairs is an excellent way to promote brand, both face-to-face and virtually. Developing CSR should also be part of marketing strategy due to its positive effects on brand preference and reputation, especially in the context the COVID-19 pandemic. Finally, social media should be part of the marketing communication agenda, particularly for younger people, who tend to trust conventional advertising less (Dwivedi & McDonald, 2018). Managers should strive to stimulate social media interaction, enhance brand preference and promote the winery’s image, by using interactive digital media, which is known to have a positive effect on brands (Canovi & Pucciarelli, 2019), such as video tasting sessions and blogs. It is important to encourage wine consumers to engage in the creation of brand content in social media communications (Godey et al., 2016; Schivinski & Dabrowski, 2015). Practitioners should consider the findings of this study when developing communication strategies to enhance brand preference and those strategies should be pretested with the target market to maximize potential benefits.

Second, managers should pay attention to the importance of brand image as a strategic tool (Ebrahim et al., 2016), that is, if their goal is to build a strong brand image capable of influencing consumer preference and of stimulating visits to wineries. Thus, managers should improve the functional, affective, and reputational attributes of their vineyards, wines, and wineries. Wine should have a consistent quality over time, and it should be presented with appropriate packaging. Wine should have a strong regional brand, and it must respond to the needs and tastes of its target market. Brand image must be an important long-term strategic tool used by managers to build stronger preferences and, thus, influence the behavioral tendency to repeat visits to wineries.

Following these recommendations will enhance intention to visit wineries. The strength of a brand indicates the importance of the wine brand for wine tourists. Enhancing wine brand preference will induce tourists to provide positive assessments of their visits to wineries. The results of this study strongly suggest that winery managers should focus their efforts on brand preference’s antecedents to improve brand communication and brand image. The findings reveal that managers should make greater investments in their communication resources and branding activities to enhance tourists’ intention to visit.

### Limitations and Future Research

This research offers significant advice to managers seeking to increase intention to visit wineries. However, when evaluating the method and findings of this study, several limitations need to be considered. First, this study is limited to wineries located in Spain. Future research could apply this theoretical framework to other wine-producing countries, which, we believe, would validate and generalize the findings. Thus, the model proposed in this research can offer a better understanding of contextual and cultural differences in consumer behavior regarding the roles of advertising promotion, sponsorship-public relations, corporate social responsibility, and social media in brand communication.

Second, we used the most widely accepted dimensions to measure brand communication and brand image, but other components do exist and could be considered in future studies. Our study contributes by increasing the understanding of the roles of advertising promotion, sponsorship-public relations, CSR, and social media in brand communication, as well as functional, emotional and reputation components in brand image development.

Third, the drivers of brand preference were limited to brand communication and brand image. Additional drivers, such as brand equity, brand knowledge (Schivinski & Dabrowski, 2015) or corporate credibility (Ebrahim et al., 2016), could be considered in future studies. In addition, intention to visit was presented as the result of brand preference. Other outcomes, such as loyalty, satisfaction, positioning, brand authenticity (Dwivedi & McDonald, 2018), or brand personality (Lee et al. 2020) could be investigated. Brand image and intention to visit have a direct relationship (Qu et al. 2011). Future research could analyze variables that moderate the relationships among the research constructs (e.g., gender, age, income, or education), bidirectional linkages or even how COVID-19 affects the use of various communication tools in wine tourism markets.

## Supplementary Information

Below is the link to the electronic supplementary material.

Supplementary file1 (DOCX 17 KB)

## Data Availability

Under request.
