# Supplementary material for: The role of novel instruments of brand communication and brand image in building consumers’ brand preference and intention to visit wineries
Source: Curr Psychol. 2022 Jan 7:1–17. Online ahead of print. doi: 10.1007/s12144-021-02656-w (PMC8740867; doi:10.1007/s12144-021-02656-w)
Supplement: Supplementary file 1 — Supplementary file1 (DOCX 17 KB) [file 12144_2021_2656_MOESM1_ESM.docx]

**Appendix. Means, Standard Deviation, Skewness and Kurtosis**

| **Variable** | **Mean** | **SD** | **Skewness** | **Kurtosis** |
| --- | --- | --- | --- | --- |
| ADV1 | 5.50 | 1.129 | -0.423 | -0.322 |
| ADV2 | 5.51 | 1.159 | -0.716 | 0.719 |
| ADV3 | 5.51 | 1.044 | -0.562 | 0.258 |
| ADV4 | 5.47 | 1.068 | -0.382 | -0.207 |
| CSR1 | 5.17 | 1.252 | -0.487 | -0.097 |
| CSR2 | 5.21 | 1.170 | -0.421 | -0.079 |
| CSR3 | 5.35 | 1.171 | -0.568 | -0.071 |
| CSR4 | 5.26 | 1.164 | -0.339 | -0.406 |
| SOC1 | 5.49 | 1.082 | -0.680 | 0.293 |
| SOC2 | 5.51 | 1.080 | -0.760 | 0.794 |
| SOC3 | 5.44 | 1.114 | -0.536 | 0.185 |
| SOC4 | 5.50 | 1.000 | -0.496 | 0.200 |
| SPO1 | 5.47 | 1.291 | -0.806 | 0.408 |
| SPO2 | 5.41 | 1.179 | -0.688 | 0.497 |
| SPO3 | 5.46 | 1.231 | -0.714 | 0.317 |
| SPO4 | 5.53 | 1.102 | -0.597 | 0.188 |
| FUN1 | 5.52 | 1.117 | -0.686 | 0.422 |
| FUN2 | 5.47 | 1.117 | -0.437 | -0.310 |
| FUN3 | 5.42 | 1.160 | -0.481 | -0.165 |
| AFF1 | 5.48 | 1.119 | -0.730 | 0.451 |
| AFF2 | 5.52 | 1.080 | -0.495 | -0.052 |
| AFF3 | 5.39 | 1.142 | -0.486 | 0.035 |
| REP1 | 5.54 | 1.200 | -0.915 | 0.832 |
| REP2 | 5.54 | 1.041 | -0.503 | 0.100 |
| PRE1 | 4.83 | 1.282 | -0.092 | -0.283 |
| PRE2 | 5.15 | 1.116 | -0.141 | -0.011 |
| PRE3 | 4.68 | 1.275 | -0.513 | 0.295 |
| INT1 | 5.41 | 1.065 | -1.053 | 2.769 |
| INT2 | 5.44 | 1.007 | -0.865 | 2.516 |
| INT3 | 5.44 | 1.028 | -1.004 | 3.136 |

Notes: ADV: advertising-promotion; SPO: sponsorship-public relations; SOC: social media; CSR: corporate social responsibility; FUN: functional image; AFF: affective image; REP: reputation; PRE: brand preference; INT: intention to visit, revisit and/or recommend wineries.
